# Lipid accumulation product is a better predictor of metabolic syndrome in Chinese adolescents: a cross-sectional study

**DOI:** 10.3389/fendo.2023.1179990

**Published:** 2023-06-23

**Authors:** Zi-yi Chen, Lei Liu, Xu-xiu Zhuang, Yi-cong Zhang, Ya-nan Ma, Yang Liu, De-liang Wen

**Affiliations:** ^1^ Institute of Health Sciences, China Medical University, Shenyang, Liaoning, China; ^2^ School of Health and Life Sciences, University of Health and Rehabilitation Sciences, Qingdao, Shandong, China; ^3^ School of Public Health, China Medical University, Shenyang, Liaoning, China

**Keywords:** lipid, lipid accumulation product, adolescent, metabolic syndrome, obesity

## Abstract

**Aim:**

Confirm and compare the degree of associations of non-traditional lipid profiles and metabolic syndrome (MetS) in Chinese adolescents, determine the lipid parameter with better predictive potential, and investigate their discriminatory power on MetS.

**Methods:**

Medical measurements, including anthropometric measurements and biochemical blood tests, were undergone among a total sample of 1112 adolescents (564 boys and 548 girls) aged from 13 to 18 years. Univariate and multivariate logistic regression analyses were applied for assessing the relationships between the levels of traditional/non-traditional lipid profiles and MetS. We performed Receiver Operating Characteristic (ROC) analyses to mensurate the effectiveness of lipid accumulation product (LAP) on the diagnosis of MetS. Meanwhile, areas under the ROC curve and the cut-off values were calculated for MetS and its components.

**Results:**

Univariate analysis showed that all our lipid profiles were closely associated with MetS (P< 0.05). LAP index showed the closest association with MetS than the other lipid profiles. Additionally, ROC analyses indicated that the LAP index showed sufficient capabilities to identify adolescents with MetS and its components.

**Conclusion:**

The LAP index is a simple and efficient tool to identify individuals with MetS in Chinese adolescents.

## 1 Introduction

Metabolic syndrome (MetS), also known as syndrome X, is a complicated multifactorial disease with insulin resistance (IR) as the central feature, characterized by central obesity, abnormal glucose regulation, hypertension, and dyslipidemia (1, 2). The upstream MetS imply a prophetic potential enabling the subsequent diseases to be alerted. Previous studies have elucidated that MetS is associated with a higher risk of type 2 diabetes mellitus (T2DM) and cardiovascular disease (CVD), the significant causes of human morbidity and mortality (3, 4). Although it is not an absolute risk indicator, MetS have been proved to predispose adolescents to have twice or more the risk of CVD and T2DM in adulthood as adolescents without MetS (5, 6). The risk over a lifetime undoubtedly will be even higher. With the increment in childhood obesity, the prevalence of MetS in juveniles in the United States stepped-up from 4.3% in 1994 to 9.8% in 2016 (7, 8). In China, the percentage was 4.1% among minors aged 10 to 16 from 7 provinces in 2012 (9). Foreseeably, the rocketing prevalence of MetS may pose a threat to public health severely. Thus, further exploration should focus on identifying the MetS population as early as possible in adolescence.

Dysregulation of lipid homeostasis is now well accepted as a common characteristic of many diseases, especially metabolic diseases, and the alterations of lipid profiles may precede the occurrence of diseases (10, 11). Studies have confirmed that traditional lipid profiles are associated with MetS, and exhibit predictive abilities to some extent (12). Some innovative reports suggested that compared with traditional ones, the calculated non-traditional lipid profiles are better predictors for MetS (13–15), which may result from the fact that changes in newly calculated lipid parameters integrate the occurrence of multiple traditional lipid abnormalities. Additionally, the non-traditional lipid profiles do not require a high expenditure of time and expense, are more convenient and affordable detecting tools. These studies provide the possibility for early and expediently clinical diagnosing of MetS. But despite multifarious calculated non-traditional lipid profiles being studied, the most suitable parameters for clinical application have not been determined.

Hereby, this study aims to estimate the associations between non-traditional lipid profiles and MetS, and find the one with the most potential. To investigate the discriminating power of promising parameter for MetS, and provide the optimal cut-off value for reference.

## 2 Materials and methods

### 2.1 Study population

The study was carried out in two middle schools in Shenyang from August 2018 to March 2019. Students with restricted physical activity or (and) secondary obesity brought on by genetic diseases, congenital heart disease, endocrine abnormalities, etc. were excluded. After applying exclusion criteria, 1238 students were enrolled in our study. Afterward, participants whose ages were not specified (n = 58), or (and) did not participate in anthropometry (n = 65), or (and) were unable to obtain reports of biochemical blood tests (n = 1), or (and) duplicate data (n = 2) were excluded from our study. Data of the remaining 1112 individuals were used for further measurements.

### 2.2 Physical examinations

Physical examinations were performed following the standards of World Health Organization (16). Height of the subjects was measured to one decimal place (centimeter) by portable rangefinder (SECA 213, Hamburg, Germany), and weight was measured to kilograms with a body fat analysis meter (TANITA DC430MA, Tokyo, Japan). Neck girths, waistlines, hiplines, and thigh girths were measured manually with a soft ruler, respectively.

### 2.3 Blood sample collection and assessments

Intravenous blood samples were collected and preserved after 12 hours fasting using blood collection tubes containing EDTA (Becton Dickinson and Co., UK). The collected samples were directly centrifuged at 4°C for 10 min at a speed of 3000 rpm and all EDTA-plasma was aliquot and kept at -80°C until tested simultaneously. The plasma content of triglyceride (TG), cholesterol (TC), high-density lipoprotein cholesterol (HDL-C), low-density lipoprotein cholesterol (LDL-C), apolipoprotein A1 (apoA1) and B (apoB), fasting plasma glucose (FPG), aspartate aminotransferase (AST), alanine aminotransferase (ALT), alkaline phosphatase (ALP), γ-glutamyl transferase (GGT), and total bilirubin (BILT) were detected by an automatic biochemistry analyzer (ARCHITECT c1600, Japan).

### 2.4 Definition

MetS were diagnosed based on the International Diabetes Federation consensus report (17), combined with the items involved in this physical examination. In our study, we conducted an on-campus survey of 13 to 18-year-olds. Blood pressure was not measured, thus MetS were identified when any two of the following three factors are attached to central obesity (1): fasting plasma glucose (FPG) level above 5.6 mmol/L or known T2DM; (2) TG level of more than 150 mg/dL; (3) HDL-C level less than 40 mg/dL in boys of all ages and girls under 16, and less than 50 mg/dL for girls older than or equal to 16 years of age. Central obesity is defined as a child under 16 years of age with a waist circumference at or above the 90th percentile of children of the same age and sex, and a waist circumference greater than or equal to 90 cm and 80 cm for male and female, respectively, after 16 years old.

### 2.5 Statistical analysis

The non-traditional lipid parameters evaluated in the present study included TG/HDL-C, TC/HDL-C, LDL-C/HDL-C, non-HDL-C, atherogenic (ATH) index, lipoprotein combine index (LCI), apoB/apoA1, HDL-C/apoA1, LDL-C/apoB, lipid accumulation product (LAP), and visceral adiposity index (VAI). Continuous data were displayed with the median and interquartile range (upper quartile and lower quartile). Since the data distribution in this study is non-normal according to the Kolmogorov-Smirnov test (*P*< 0.05), we adopted the Mann-Whitney U test to compare participants’ general characteristics with and without MetS. Pearson chi-square was utilized to analyze the association between categorical variables. To eliminate the deviation caused by dimension and thus contribute to the comparisons of the associations between lipid profiles and MetS, each variable was standardized by the zero-mean normalization before logistic regression analyses. Univariate and multivariate logistic regression after adjusting for confounders were used to evaluate the associations between lipid profiles and the risk of MetS, and the adjusted odds ratio (OR) was presented with 95% confidence interval (95% CI). Additionally, we computed the area under the receiver operating characteristic (ROC) curve (AUC) for parameters with stable relevance to evaluate their resolving abilities in the detection of MetS and also determined their optimum cut-off values by the Yoden index. Statistical analyses were all conducted by SPSS 23.0 (IBM Corp). A P value< 0.05 was regarded statistically. The equations for ATH index, LCI, LAP and VAI are shown below:


$$ATH\;index=\frac{\left(TC-HDLC\right)\times apoB}{HDLC\times apoA1}$$

$$LCI=\frac{TG\times TC\times LDLC}{HDLC}$$

LAP for boys=(Waistline−65)×TG

LAP for girls=(Waistline−58)×TG

$$VAI\;for\;boys=\frac{Waistline}{39.68+1.88\times BMI}\times \frac{TG}{1.03}\times \frac{1.31}{HDLC}$$

$$VAI\;for\;girls=\frac{Waistline}{36.58+1.89\times BMI}\times \frac{TG}{0.81}\times \frac{1.52}{HDLC}$$

### 2.6 Ethic approval

The ethics committee of the China Medical University approved this study, and each participant had informed consent (Ethics Approval No. [2020] 077). All methods were carried out under the Declaration of Helsinki and dependent guidelines.

## 3 Results

### 3.1 Basic characteristics

The prevalence of MetS among the subjects was 3.15%, and the prevalence by sex was 2.92% in boys and 3.4% in girls. According to the Pearson chi-square test, there was no significant difference in prevalence between boys and girls (P > 0.05). Most of the tested lipid profiles are higher in subjects diagnosed with MetS while HDL-C and apoA1 are lower. In girls, no group-related differences were observed in age, height, and the levels of ALP and BILT (P< 0.05). The same is true in boys with the addition of FPG. There are no sex-related differences in thigh girth, body mass index (BMI), FPG, TG, and LCI values among subjects (**Supplemental Table 1**).

### 3.2 Univariate and multiple logistic regression analyses

Univariate model was run for each predictive variable, using MetS as its outcome. Among male subjects, as illustrated in **Table 1**, from the univariate logistic regression analyses we found all lipid parameters are significantly associated with the risk of MetS. Notably, the association between TG and MetS is rivaled that between non-traditional lipid profiles and MetS. LAP (OR: 5.878, 95%CI: 3.365-10.269, P< 0.001) and HDL-C (OR: 0.138, 95%CI: 0.061-0.311, P< 0.001) were respectively the most influential negative and positive factor. Multivariate logistic regression was carried out to suppress the interference from other confounding factors. Common health metrics that are significant in univariate logistic regression (P< 0.1), including bodily circumferences, weight, BMI, and FPG, were synchronously put into model 1 with each lipid parameter. Furthermore, we incorporated conventional liver function indexes, factors with sufficient association strength with MetS (ALT, AST, and GGT) were qualified to be combined with lipid parameters to build model 2. After we threw all the steps together, LAP (OR: 17.422, 95%CI: 4.504-67.392, P< 0.001) was still the most significant negative factor, and HDL-C (OR: 0.131, 95%CI: 0.043-0.397, P< 0.001) still has the greatest positive association with MetS. The ORs of TC, LDL-C, and apoA1/HDL-C were no longer statistically significant, while the ORs of LCI, apoB/apoA1, apoB/LDL-C, and VAI increased.

**Table 1:** Univariate and multivariate regression analyses for boys.

Variables	Univariate		Model 1		Model 2	
	OR (95%CI)	P value	OR (95%CI)	P value	OR (95%CI)	P value
Traditional lipid profiles						
Zscore: TG	4.941 (3.002; 8.131)	<0.001	4.43 (2.322; 8.453)	<0.001	6.494 (2.702; 15.609)	<0.001
Zscore: TC	2.275 (1.511; 3.425)	<0.001	1.457 (0.795; 2.671)	0.223	1.482 (0.73; 3.011)	0.276
Zscore: HDL-C	0.138 (0.061; 0.311)	<0.001	0.156 (0.054; 0.449)	0.001	0.131 (0.043; 0.397)	<0.001
Zscore: LDL-C	2.248 (1.528; 3.308)	<0.001	1.57 (0.842; 2.93)	0.156	1.586 (0.779; 3.228)	0.204
Zscore: apoA1	0.367 (0.198; 0.68)	0.001	0.274 (0.122; 0.614)	0.002	0.248 (0.105; 0.586)	0.001
Zscore: apoB	2.623 (1.806; 3.812)	<0.001	2.282 (1.319; 3.95)	0.003	2.769 (1.471; 5.212)	0.002
Non-traditional lipid profiles						
Zscore: TG/HDL-C	4.692 (2.853; 7.715)	<0.001	3.857 (2.111; 7.046)	<0.001	4.784 (2.336; 9.799)	<0.001
Zscore: TC/HDL-C	4.126 (2.462; 6.913)	<0.001	2.289 (1.186; 4.418)	0.014	2.125 (1.109; 4.069)	0.023
Zscore: LDL-C/HDL-C	4.030 (2.449; 6.631)	<0.001	2.878 (1.488; 5.566)	0.002	2.665 (1.404; 5.059)	0.003
Zscore: non-HDL-C	3.183 (2.098; 4.83)	<0.001	2.489 (1.328; 4.666)	0.004	2.876 (1.403; 5.895)	0.004
Zscore: ATH index	3.881 (2.579; 5.838)	<0.001	3.724 (2.06; 6.732)	<0.001	3.79 (1.998; 7.191)	<0.001
Zscore: LCI	3.723 (2.48; 5.589)	<0.001	3.522 (2.038; 6.085)	<0.001	5.354 (2.298; 12.477)	<0.001
Zscore: apoB/apoA1	3.632 (2.356; 5.601)	<0.001	4.298 (2.184; 8.457)	<0.001	5.287 (2.246; 12.443)	<0.001
Zscore: HDL-C/apoA1	0.251 (0.139; 0.452)	<0.001	0.426 (0.217; 0.834)	0.013	0.4 (0.202; 0.794)	0.009
Zscore: LDL-C/apoB	0.533 (0.372; 0.763)	0.001	0.483 (0.321; 0.726)	<0.001	0.392 (0.242; 0.646)	<0.001
Zscore: LAP	5.878 (3.365; 10.269)	<0.001	10.263 (3.639; 28.95)	<0.001	17.422 (4.504; 67.392)	<0.001
Zscore: VAI	4.609 (2.849; 7.457)	<0.001	4.082 (2.197; 7.584)	<0.001	5.219 (2.423; 11.242)	<0.001

Model 1: adjustment risk factors including circumferences, weight, BMI, FPG; Model 2: Variables (circumferences, weight, BMI, FPG, ALT, AST, GGT) that were significant (P<0.05) in univariate models were simultaneously entered into this multivariable model.

Similar methods were used for female subjects (**Table 2**). In univariate logistic regression analysis, both traditional and non-traditional lipid profiles showed significant associations with MetS, among which the HDL-C (OR: 0.043, 95%CI: 0.014-0.133, P< 0.001) and LAP (OR: 23.135, 95%CI: 3.403-157.3, P = 0.001) is the most significant positive and negative factor, respectively. Bodily circumferences, height, weight, BMI, and FPG were incorporated to build model 1. Whereafter, the ORs of TC, TG/HDL-C, and VAI were not statistically significant anymore (P > 0.05). In model 2, liver functions including ALT, AST, and GGT were added as confounding factors. The associations between LDL-C, apoB, non-HDL-C, and MetS were no longer statistically significant factors (P > 0.05). Among the remaining indicators, associations between TG, HDL-C, apoA1, apoB/apoA1, HDL-C/apoA1, LAP, and MetS enhanced obviously. The OR values of TC/HDL-C, LDL-C/HDL-C, ATH index, and LDL-C/apoB were relatively stable, while the OR value of LCI decreased.

**Table 2:** Univariate and multivariate regression analyses for girls.

Variables	Univariate		Model 1		Model 2	
	OR (95%CI)	P value	OR (95%CI)	P value	OR (95%CI)	P value
Traditional lipid profiles						
Zscore: TG	5.649 (3.325; 9.599)	<0.001	17.789 (3.922; 80.693)	<0.001	13.362 (2.878; 62.028)	0.001
Zscore: TC	2.208 (1.423; 3.423)	<0.001	1.435 (0.743; 2.772)	0.283	0.898 (0.421; 1.916)	0.78
Zscore: HDL-C	0.043 (0.014; 0.133)	<0.001	0.005 (<0.001; 0.080)	<0.001	0.001 (<0.001; 0.114)	0.006
Zscore: LDL-C	3.015 (1.893; 4.801)	<0.001	1.937 (0.947; 3.961)	0.07	1.193 (0.54; 2.636)	0.663
Zscore: apoA1	0.253 (0.132; 0.486)	<0.001	0.101 (0.027; 0.382)	0.001	0.027 (0.003; 0.25)	0.001
Zscore: apoB	3.529 (2.222; 5.605)	<0.001	2.865 (1.561; 5.258)	0.001	2.03 (0.938; 4.394)	0.072
Non-traditional lipid profiles						
Zscore: TG/HDL-C	9.427 (4.473; 19.868)	<0.001	3.029 (<0.001; 1.140)	0.442	1.06E + 72 (0; —)	0.925
Zscore: TC/HDL-C	11.264 (4.976; 25.499)	<0.001	12.89 (3.684; 45.102)	<0.001	11.729 (3.16; 43.532)	<0.001
Zscore: LDL-C/HDL-C	8.607 (4.311; 17.182)	<0.001	10.093 (3.344; 30.458)	<0.001	8.651 (2.733; 27.38)	<0.001
Zscore: non-HDL-C	4.595 (2.695; 7.837)	<0.001	3.036 (1.409; 6.543)	0.005	1.866 (0.803; 4.338)	0.147
Zscore: ATH index	5.125 (3.148; 8.344)	<0.001	5.648 (2.519; 12.664)	<0.001	5.251 (2.238; 12.322)	<0.001
Zscore: LCI	4.682 (2.965; 7.392)	<0.001	4.317 (2.316; 8.044)	<0.001	3.715 (1.847; 7.47)	<0.001
Zscore: apoB/apoA1	5.335 (3.150; 9.035)	<0.001	7.218 (2.782; 18.731)	<0.001	6.135 (2.27; 16.581)	<0.001
Zscore: HDL-C/apoA1	0.062 (0.024; 0.162)	<0.001	0.026 (0.004; 0.192)	<0.001	0.023 (0.002; 0.236)	0.001
Zscore: LDL-C/apoB	0.388 (0.241; 0.626)	<0.001	0.351 (0.205; 0.602)	<0.001	0.348 (0.192; 0.63)	0.001
Zscore: LAP	3.818 (2.572; 5.666)	<0.001	35.154 (5.519; 223.906)	<0.001	23.135 (3.403; 157.3)	0.001
Zscore: VAI	7.411 (3.938; 13.949)	<0.001	4.311E + 11 (0; 3.698E + 40)	0.431	2.005E + 89 (0; —)	0.929

Model 1: adjustment risk factors including circumferences, height, weight, BMI, FPG; Model 2: Variables (circumferences, height, weight, BMI, FPG, ALT, AST, GGT) that were significant (P<0.05) in univariate models were simultaneously entered into this multivariable model.

We found that the associations between MetS and negative factors in female subjects were generally higher than that in males. LAP was the most closely associated negative factor with MetS in each sex.

### 3.3 Diagnostic value of LAP in detecting MetS

To evaluate the predictive value of LAP, the most remarkable parameter in logistic regression analyses, we drew the receiver operating characteristic curves (ROC) of MetS and its components based on LAP and calculated the areas under the curve (AUC). As shown in the ROC analyses (**Table 3**), the LAP ratio occupies outstanding prediction performance for MetS (girls: AUC = 0.964, boys: AUC = 0.99). Except for MetS, LAP was also an excellent predictor for high TG (girls: AUC = 0.959, boys: AUC = 0.97), central obesity (girls: AUC = 0.95, boys: AUC = 0.959) and effectual predictor for low HDL-C (girls: AUC = 0.743, boys: AUC = 0.718), high FPG (girls: AUC = 0.785, boys: AUC = 0.755). The cut-off values in detecting MetS are 38.025 and 28.91 in boys and girls, respectively. Our results suggested that LAP can identify each component of MetS and exhibited sufficient capacity to distinguish the MetS patients in the Chinese adolescent population.

**Table 3:** AUC of the ROC curve of LAP to predict MetS and its components in adolescents.

Variables	Boys					Girls				
	Cut-off	Sensitivity	Specificity	AUC (95%CI)	P value	Cut-off	Sensitivity	Specificity	AUC (95%CI)	P value
Central Obesity	13.31	0.878	0.902	0.959 (0.944-0.973)	<0.001	12.740	0.887	0.861	0.95 (0.933-0.967)	<0.001
High FPG	25.328	0.667	0.836	0.755 (0.575-0.936)	0.009	28.910	0.667	0.871	0.785 (0.65-0.921)	0.001
High TG	25.925	0.966	0.875	0.97 (0.947-0.994)	<0.001	23.080	0.960	0.859	0.959 (0.929-0.99)	<0.001
Low HDL-C	13.523	0.619	0.779	0.743 (0.693-0.793)	<0.001	9.110	0.777	0.584	0.718 (0.661-0.775)	<0.001
MetS	38.025	1	0.936	0.99 (0.98-0.999)	<0.001	28.910	1	0.889	0.964 (0.944-0.983)	<0.001

## 4 Discussion

### 4.1 Summary

In the current study, we confirmed that the LAP levels increased in adolescents with MetS compared with normal subjects. Among the 11 common non-traditional lipid profiles we selected, LAP showed the strongest association with MetS. In agreement with previous studies, LAP has been confirmed to identify MetS independently and exhibited sufficient capacity to identity the components of MetS (18–22). Current research suggests that MetS develops after an accumulation of abdominal fat, and dyslipidemia precedes the occurrence of MetS. Therefore, LAP may have an ability to predict MetS in the early stages (23).

In addition, we reported the associations between lipid profiles and MetS after adjusting for significant common health metrics and liver function indexes. Although the non-traditional lipid profiles generally displayed a superior association with MetS, a couple of traditional lipid profiles have preeminent performances. Moreover, association with MetS was found to be reduced for some calculated parameters after the combination of these potent individual lipids. One of the possible reasons is the low prevalence in adolescent subjects resulting in the over-fitting of the model. This may reflect the non-traditional lipid profiles that integrate more than one lipid parameter are more reliable MetS predictors. TG and HDL-C, two components of MetS, are the most influential among all lipid profiles in subjects regardless of sex. Yet, there is still controversy about whether traditional lipid profiles can be used as MetS predictors and it is biased to regard them as powerful MetS predictors (24–26).

### 4.2 Strengths and limitations

Our study had a sufficient sample size, a balanced sex ratio, and kept as many eligible participants as possible. There are also several limitations to our study. Firstly, the MetS patients are few and may not be sufficient to represent the overall prevalence level in adolescents. Secondly, our cross-sectional study did not evaluate the long-term relationship between MetS and lipid profile levels, and prospective follow-up studies are needed. Thirdly, the diagnostic criteria of MetS have not been clearly defined yet, and the criteria we use may be different from other reports. Fourthly, there may be MetS patients who have not been diagnosed because we did not take blood pressure measurements. Besides, the prevalence of hypertension varies among MetS patients with different ages and sexes. Excluding hypertension from the components of MetS may affect the future design of specific treatments for different populations (27). Lastly, only Chinese adolescents were involved in our study and their ethnicity was not collected. Besides, the study sites are confined to Shenyang.

### 4.3 Comparison with existing literature

So far, only a few data concerning the association of LAP on MetS risk in Chinese, especially among adolescents. Cut-off values of non-traditional lipid profiles to identify MetS patients are rarely reported, and the majority of studies available only involved a specific population. Our study extends the current findings by proposing the cut-off values of LAP to distinguish individuals with MetS in Chinese adolescents. Our result differs from the data for Chinese elders (28), Indian PCOS women (29), and Iran adults (30), but close to a large community-based and multi-ethnic population study in China (31). These demonstrated that a single cut-off value is not adequate for a diverse population due to various ethnicity and age.

### 4.4 Implications for research and practice

Although the development of MetS is not entirely understood, existing studies believe that IR derived from central obesity is the most critical pathogenesis of MetS. Dyslipidemia leads to the accumulation of lipids and their metabolites, which impair the function of B cell, induce the occurrence of IR, then facilitate the development of MetS components, and finally leads to the occurrence of MetS (32). The LAP index has been considered a surrogate indicator of central obesity (33) and IR (34–37) formerly, which also showed an apparent association with MetS risk in our study. On one aspect, the calculation formula of the LAP index contains TG, which makes it possible to reflect dyslipidemia and MetS directly. On the other hand, LAP is generally regarded as an indicator in measuring visceral fat, while central obesity is another marker of ectopic fat accumulation (38) as a component of MetS. Accordingly, the role of LAP in metabolic diseases has been gradually gaining attention in late years. Researchers found that LAP level was positively correlated with an increased risk of long-term cardiovascular disease in populations with or without pre-existing CVD (39–41). In several large population-based studies, LAP was considered a powerful risk predictor for incident diabetes and capable of being used as a diagnostic tool (42–44). Some studies have also indicated that LAP is significantly associated with hypertension, but most of the current studies are concentrated in East Asia, and the data on different ethnic populations are temporarily lacking (45, 46).

An overview of the patient’s condition is needed to diagnose MetS, which is complicated in clinical practice. Nonetheless, levels of most lipid profiles are measured routinely and the non-traditional lipid ratios can be calculated easily. The strengths of this study are that we enumerated most of the non-traditional lipid profiles that are now extensively studied and made comparisons to help ascertain the most reliable tool for physicians to identify patients with MetS.

## Data availability statement

The raw data supporting the conclusions of this article will be made available by the authors, without undue reservation.

## Ethics statement

The studies involving human participants were reviewed and approved by the ethics committee of the China Medical University. Written informed consent to participate in this study was provided by the participants’ legal guardian/next of kin.

## Author contributions

Z-YC, LL: research design, data collection, data analysis, manuscript writing. Z-YC, LL, X-XZ, Y-CZ: blood biochemical measurements, data collection. YL: project development, data collection. YL, Y-NM, D-LW: data interpretation, manuscript revise. All authors contributed to the article and approved the submitted version.
