# Supplementary material for: Lipid accumulation product is a better predictor of metabolic syndrome in Chinese adolescents: a cross-sectional study
Source: Front Endocrinol (Lausanne). 2023 Jun 23;14:1179990. doi: 10.3389/fendo.2023.1179990 (PMC10326626; doi:10.3389/fendo.2023.1179990)
Supplement: Supplementary file 1 [file Table_1.docx]

Supplementary Table 1 Demographic characteristics of study participants with or without MetS.

| Variables | Boys (n = 564) |  |  |  | Girls (n = 548) |  |  |  | *P* value |
| --- | --- | --- | --- | --- | --- | --- | --- | --- | --- |
|  | MetS (n = 16) | non-MetS (n = 548) | *P* value |  | MetS (n = 18) | non-MetS (n = 530) | *P* value |  |  |
| Age (years) | 15 (15; 15) | 15 (15;16) | 0.062 |  | 15 (15; 16) | 15 (14;16) | 0.966 |  | 0.001 |
| Neck girth (cm) | 41 (40; 41.6) | 36 (34; 38) | <0.001 |  | 36.95 (35; 38.13) | 32 (31; 34) | <0.001 |  | <0.001 |
| Waistline (cm) | 101.5 (94.13; 108.5) | 75 (70; 83.38) | <0.001 |  | 88.5 (82.75; 93.38) | 70 (64.9; 77) | <0.001 |  | <0.001 |
| Hipline (cm) | 115 (110.25; 116) | 96.5 (91; 102) | <0.001 |  | 106 (99.5; 113) | 95.15 (90; 101) | <0.001 |  | 0.002 |
| Thigh girth (cm) | 66.05 (61.25; 71.25) | 54.5 (51; 61) | <0.001 |  | 64.2 (57.9; 69.2) | 55 (51; 60) | <0.001 |  | 0.808 |
| Height (m) | 175 (172.3; 179.93) | 175 (170.25; 178.28) | 0.432 |  | 160.45 (156.6; 164.25) | 162.7 (159.08; 166.93) | 0.072 |  | <0.001 |
| Weight (kg) | 96.4 (86.63; 106.7) | 64.15 (56.55; 75.48) | <0.001 |  | 77 (71.53; 94.43) | 55 (49.45; 64.6) | <0.001 |  | <0.001 |
| BMI (kg/m^2^) | 30.81 (29.17; 34.5) | 21.22 (18.8; 24.72) | <0.001 |  | 31.53 (28.23; 35.73) | 20.8 (18.67; 24.1) | <0.001 |  | 0.212 |
| FPG (mmol/L) | 4.69 (4.01; 5.4) | 4.34 (4.02; 4.66) | 0.131 |  | 4.88 (4.08; 8.36) | 4.28 (4.04; 4.65) | 0.037 |  | 0.589 |
| ALT (U/L) | 39 (19; 52.5) | 14(10; 20) | <0.001 |  | 32 (11; 44.75) | 10 (8; 14) | <0.001 |  | <0.001 |
| AST (U/L) | 22.5 (18; 30) | 17 (15; 21) | 0.005 |  | 22 (15.75; 25.5) | 15 (14; 18) | <0.001 |  | <0.001 |
| ALP (U/L) | 120.5 (97; 161.25) | 118 (94; 156.75) | 0.871 |  | 89 (73.75; 105.5) | 79 (67; 95) | 0.117 |  | <0.001 |
| GGT (U/L) | 32.5 (24.75; 41) | 18 (15; 24) | <0.001 |  | 27 (23; 41.5) | 14 (12; 17) | <0.001 |  | <0.001 |
| BILT (μmol/L) | 11.8 (7.28; 15.1) | 12.65 (9.8; 17.2) | 0.219 |  | 8.2 (6.8; 12.15) | 10.1 (7.8; 13.33) | 0.127 |  | <0.001 |
| **Traditional lipid profile** | | | |  |  |  |  |  |  |
| TG (mg/dL) | 193.98 (162.98; 243.8) | 63.77 (45.39; 84.81) | <0.001 |  | 168.29 (118.02; 207.93) | 64.66 (47.61; 88.57) | <0.001 |  | 0.301 |
| TC (mg/dL) | 164.73 (162.98; 243.8) | 141.92 (126.06; 157.39) | <0.001 |  | 180.97 (171.89; 189.48) | 153.13 (137.18; 171.31) | <0.001 |  | <0.001 |
| HDL-C (mg/dL) | 34.61 (30.26; 38.28) | 47.18 (41.38; 53.75) | <0.001 |  | 37.12 (34.61; 39.54) | 55.3 (47.18; 63.42) | <0.001 |  | <0.001 |
| LDL-C (mg/dL) | 106.73 (87.01; 117.75) | 79.27 (65.74; 92.03) | <0.001 |  | 111.56 (103.83; 123.74) | 83.53 (69.22; 98.22) | <0.001 |  | <0.001 |
| apoA1 (mg/dL) | 105 (95.75; 123.25) | 120 (110;133) | 0.002 |  | 114 (105.5; 123.25) | 134 (122; 149) | <0.001 |  | <0.001 |
| apoB (mg/dL) | 82 (75; 92.5) | 57 (49; 67) | <0.001 |  | 86 (81.5; 98.75) | 59 (50; 70) | <0.001 |  | 0.011 |
| **Nontraditional lipid profiles** | | | |  |  |  |  |  |  |
| TG/HDL-C | 5.68 (5.12; 7.3) | 1.33 (0.91; 2) | <0.001 |  | 4.82 (3.22; 6.18) | 1.13 (0.79; 1.73) | <0.001 |  | 0.001 |
| TC/HDL-C | 4.9 (4.78; 5.68) | 2.95 (2.55; 3.46) | <0.001 |  | 4.71 (4.37; 5.4) | 2.71 (2.37; 3.28) | <0.001 |  | <0.001 |
| LDL-C/HDL-C | 3.06 (2.91; 3.49) | 1.67 (1.33; 2.07) | <0.001 |  | 2.97 (2.6; 3.45) | 1.48 (1.18; 1.92) | <0.001 |  | <0.001 |
| non-HDL-C (mg/dL) | 134.38 (117.85; 149.07) | 92.42 (78.89; 108.47) | <0.001 |  | 143.85 (134.57; 153.71) | 95.71 (80.82; 114.56) | <0.001 |  | 0.006 |
| ATH index | 3.18 (2.52; 4.26) | 0.94 (0.62; 1.38) | <0.001 |  | 2.86 (2.32; 3.71) | 0.75 (0.4; 1.18) | <0.001 |  | <0.001 |
| LCI | 30.92 (23.05; 38.21) | 4.21 (2.58; 7.09) | <0.001 |  | 25.53 (21.89; 33.58) | 4.13 (2.55; 7.31) | <0.001 |  | 0.918 |
| apoB/apoA1 | 0.83 (0.69; 0.89) | 0.48 (0.39; 0.57) | <0.001 |  | 0.77 (0.68; 0.87) | 0.43 (0.36; 0.54) | <0.001 |  | <0.001 |
| HDL-C/apoA1 | 0.32 (0.29; 0.35) | 0.39 (0.36; 0.43) | <0.001 |  | 0.33 (0.31; 0.34) | 0.41 (0.38; 0.44) | <0.001 |  | <0.001 |
| LDL-C/apoB | 1.27 (1.17; 1.3) | 1.38 (1.31; 1.44) | <0.001 |  | 1.28 (1.21; 1.35) | 1.4 (1.34; 1.46) | <0.001 |  | <0.001 |
| LAP | 88.94 (70.51; 101.2) | 6.86 (2.6; 15.64) | <0.001 |  | 58.56 (39.63; 79.14) | 8.48 (4.07; 16.48) | <0.001 |  | 0.002 |
| VAI | 3.33 (2.74; 3.99) | 0.71 (0.48; 1.07) | <0.001 |  | 3.7 (2.59; 4.7) | 0.85 (0.6; 1.33) | <0.001 |  | <0.001 |
